# Case Report: Combined umbilical cord blood and peripheral blood stem cell transplantation with donor lymphocyte infusion for R/R AML post CAR-CLL1 failure

**DOI:** 10.3389/fimmu.2025.1598754

**Published:** 2025-06-05

**Authors:** Fanqiao Meng, Yu Liu, Dongfeng Zeng

**Affiliations:** ^1^ Department of Hematology, Daping Hospital, Third Military Medical University (Army Medical University), Chongqing, China; ^2^ Division of Daping Hospital, Chongqing Key Laboratory of Hematology and Microenvironment, Chongqing, China

**Keywords:** umbilical cord blood, allogeneic hematopoietic stem cell transplantation, donor lymphocyte infusion, acute myeloid leukemia, CAR-CLL1

## Abstract

Relapsed/refractory acute myeloid leukemia (R/R AML) carries an extremely poor prognosis, particularly in patients who fail chimeric antigen receptor T-cell (CAR-T) therapy, with no effective treatment options currently available. We report a 35-year-old male with AML who experienced relapse after multiple lines of high-intensity chemotherapy. Salvage CAR-CLL1 therapy was administered, but the patient failed to achieve hematopoietic recovery or immune reconstitution, followed by rapid disease relapse within one month and progression to septic shock. At this critical juncture, conventional therapies proved insufficient. Allogeneic hematopoietic stem cell transplantation (allo-HSCT) includes peripheral blood stem cell transplantation (PBSCT) and umbilical cord blood (UCB). The patient underwent combined UCB and PBSCT with donor lymphocyte infusion (DLI). He has since achieved sustained remission, though developed cutaneous and intestinal graft-versus-host disease (GVHD), which is currently under control. This case highlights that combined UCB and PBSCT with DLI may represent a potential therapeutic option for R/R AML following CAR-T therapy failure, warranting further investigation in similar high-risk scenarios.

## Introduction

Relapsed/refractory acute myeloid leukemia (R/R AML) carries a dismal prognosis (1). Chimeric antigen receptor T-cell (CAR-T) therapy is an immunotherapy that targets cancer cells by genetically engineering T cells (2). CAR-T cells specifically bind to the target antigens (such as CD19, BCMA and CLL1 expressed on tumor cells) on the surface of cancer cells through the antigen-binding region of the CAR, thereby achieving targeted killing of tumor cells (3). Despite ongoing efforts to explore novel therapies, including immune-targeted treatments, CAR-T therapy, and salvage allogeneic hematopoietic stem cell transplantation (allo-HSCT) following intensive chemotherapy, these patients still urgently require innovative strategies to improve outcomes (4, 5). In this case report, we present a 35-year-old male with R/R AML who experienced disease progression after CAR-CLL1. The patient was successfully salvaged through a combined peripheral blood stem cell transplantation (PBSCT) and umbilical cord blood (UCB), followed by donor lymphocyte infusion (DLI). This case underscores the potential of a therapeutic approach for high-risk R/R AML patients failing CAR-T therapy.

## Case presentation

**Figure 1:**
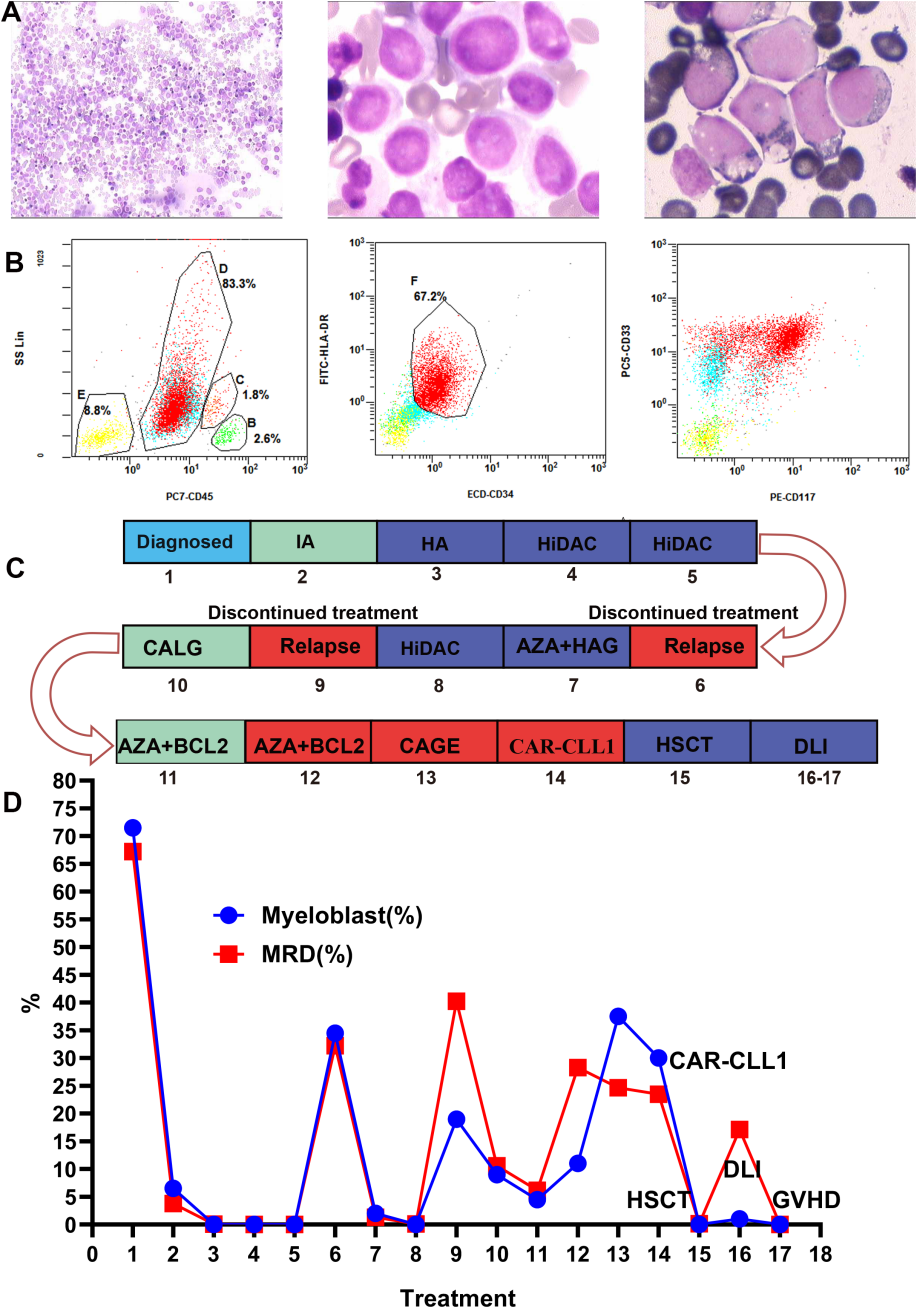
Bone marrow morphology, minimal residual disease (MRD), the timeline of treatment and efficacy evaluation. **(A)** Displays the bone marrow morphology at diagnosis, revealing myeloblasts accounting for 71.5% of nucleated cells. **(B)** Shows flow cytometry results of the bone marrow at diagnosis: 67.2% of nucleated cells resided in an abnormal region, expressing MPO, CD13, CD33, CD34, HLA-DR, CD117, and CD7, while lacking cCD3, cCD79a, CD56, CD19, CD15, and CD11b. These aberrant myeloid blasts supported the diagnosis of AML. **(C, D)** Timeline of the patient’s treatment and bone marrow assessment. Numbers in the graph represent patient treatment events. 1, Diagnosis;2, IA;3, HA;4-5, HiDAC;6, Recurrence;7, AZA+HAG;8, HiDAC;9, Recurrence;10, CALG;11-12, AZA+BCL-2;13, CAGE chemotherapy; 14, CLL1 CAR-T;15, HSCT;16-17, DLI. IA, idarubicin + cytarabin; HA, homoharringtonine + cytarabine; AZA + HAG, azacytidine +homoharringtonine + cytarabine +granulocyte colony-stimulating factor; HiDAC, high-dose cytarabine; BCL-2, venetoclax; AZA, azacytidine; CLAG, cladribine, cytarabine, granulocyte colony-stimulating factor; CAGE, cytarabine, aclarubicin, granulocyte colony-stimulating factor, etoposide; HSCT, hematopoietic stem cell transplantation; DLI, donor lymphocyte infusion; GVHD, graft-versus-host disease.

A 35 years old male presented with epistaxis in December 2022. Initial blood tests revealed significant leukocytosis (35.83×10^9^/L), moderate anemia (106 g/L), and severe thrombocytopenia (7×10^9^/L), raising clinical suspicion of leukemia. Bone marrow aspiration demonstrated markedly hypercellular marrow with predominance of granulocytic lineage (86.5%), erythroid hypoplasia (9.5%), and a 71.5% myeloblast (**Figure 1A**). Flow cytometry identified an abnormal myeloid blast population (67.2% of nucleated cells) expressing MPO, CD13, CD33, CD34, HLA-DR, CD117, and CD7 (**Figure 1B**). These findings were consistent with a diagnosis of AML. Cytogenetic analysis revealed a normal male karyotype: 46, XY (20). Next-generation sequencing (NGS) targeting 56 myeloid-related genes (**Supplementary Table S1**) detected multiple pathogenic mutations, including biallelic CEBPA mutations, an NRAS mutation, and additional mutations in GATA2 and WT1(**Supplementary Table S2**). Final diagnosis: AML with biallelic CEBPA mutations, accompanied by concurrent NRAS, GATA2, and WT1 mutations.

The patient’s treatment process and efficacy evaluation are shown in **Figures 1C, D**. In December 2022, the patient underwent IA regimen (idarubicin + cytarabine). Post-chemotherapy bone marrow evaluation revealed 6.5% myeloblasts and minimal residual disease (MRD) of 3.8% by flow cytometry. In January 2023, consolidation therapy with HA (homoharringtonine + cytarabine) achieved complete morphological remission with MRD reduced to 0.06%. Subsequently, two courses of high-dose cytarabine (HiDAC) chemotherapy were administered between March and April 2023. Post-treatment assessments demonstrated complete morphological remission (0% blasts) and undetectable MRD (0%).

**Figure 2:**
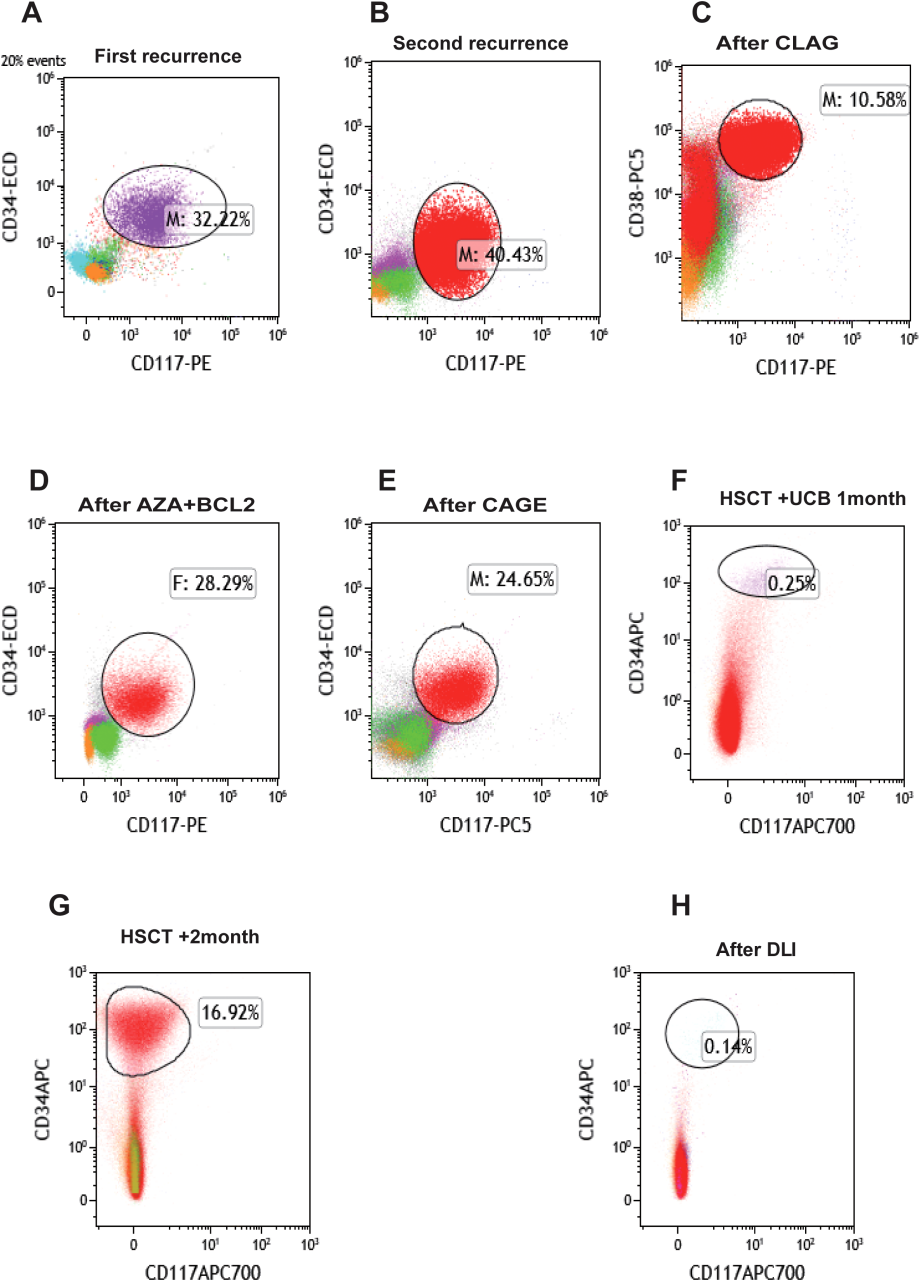
Changes of bone marrow MRD in patients during treatment. **(A)** The patient terminated autologous stem cell collection due to novel coronavirus infection, was discharged automatically, and had the first recurrence. **(B)** The patient was discharged automatically after subsequent remission with a second recurrence. **(C)** MRD status after CLAG chemotherapy. **(D)** MRD after AZA+BCL2 chemotherapy. **(E)** MRD after CAGE chemotherapy. **(F)** MRD status after HSCT+UCB treatment. **(G)** MRD status of patients after transplantation +1 month later. **(H)** MRD status after DLI treatment. BCL-2, venetoclax; AZA, azacytidine; CLAG, cladribine, cytarabine, granulocyte colony-stimulating factor; CAGE, cytarabine, aclarubicin, granulocyte colony-stimulating factor, etoposide; HSCT, hematopoietic stem cell transplantation; DLI, donor lymphocyte infusion; GVHD, graft-versus-host disease; UCB, umbilical cord blood; MRD, minimal residual disease.

Due to the lack of a suitable donor, autologous stem cell transplantation was planned. However, stem cell collection was halted after the patient tested positive for severe acute respiratory syndrome coronavirus 2. The patient discontinued treatment and self-discharged until August 2023, when relapse was confirmed (34.5% myeloblasts, MRD 32.22%, **Figure 2A**). Salvage therapy with azacitidine (AZA) + HAG (homoharringtonine + cytarabine +granulocyte colony-stimulating factor) decreased myeloblasts to 2% (MRD 1.3%). Following the completion of HiDAC in September 2023, the morphological examination showed 0% blasts with an MRD of 0.09%. Despite this, the patient again discontinued treatment and self-discharged. Relapse was subsequently confirmed in five months later (19% myeloblasts, MRD 40.43%. **Figure 2B**). February 2024, CLAG (cladribine, cytarabine, granulocyte colony-stimulating factor) therapy reduced myeloblasts to 9% (MRD 10.58%, **Figure 2C**). March 2024, AZA +venetoclax (BCL-2) therapy reduced myeloblasts to 4.5% (MRD 6.17%). But subsequent AZA +BCL-2 therapy (April 2024) failed, leading to disease progression (11% myeloblasts, MRD 28.29%, **Figure 2D**). May 2024, CAGE (cytarabine, aclarubicin, granulocyte colony-stimulating factor, etoposide) was administered, but myeloblasts further increased to 37.5% (MRD 24.65%, **Figure 2E**).

**Figure 3:**
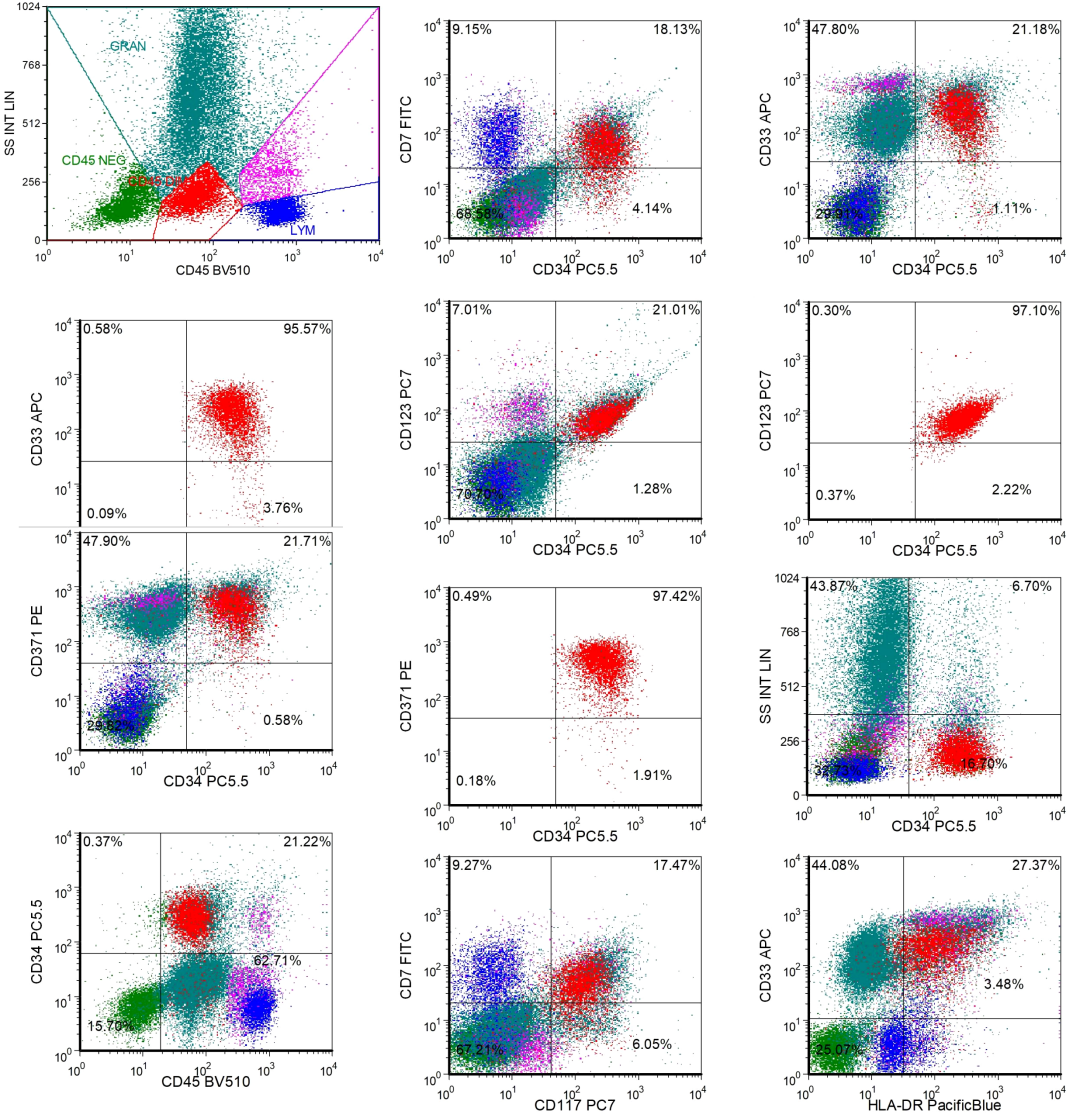
The expression of CLL1 on bone marrow AML cells of patients before CLL1 CAR-T treatment. Approximately 16.7% of AML tumor cells were observed in the bone marrow specimen submitted for examination. The results of additional CD tests are as follows: CD371+, CD33+, CD123+, HLA-DR+, CD117+, CD34+, CD45+, CD7+; CD371+ cells accounted for approximately 97.4% of the total CD34+ cells; CD33+ cells accounted for approximately 95.6% of the total CD34+ cells; CD123+ cells accounted for approximately 97.1% of the total CD34+ cells.

**Figure 4:**
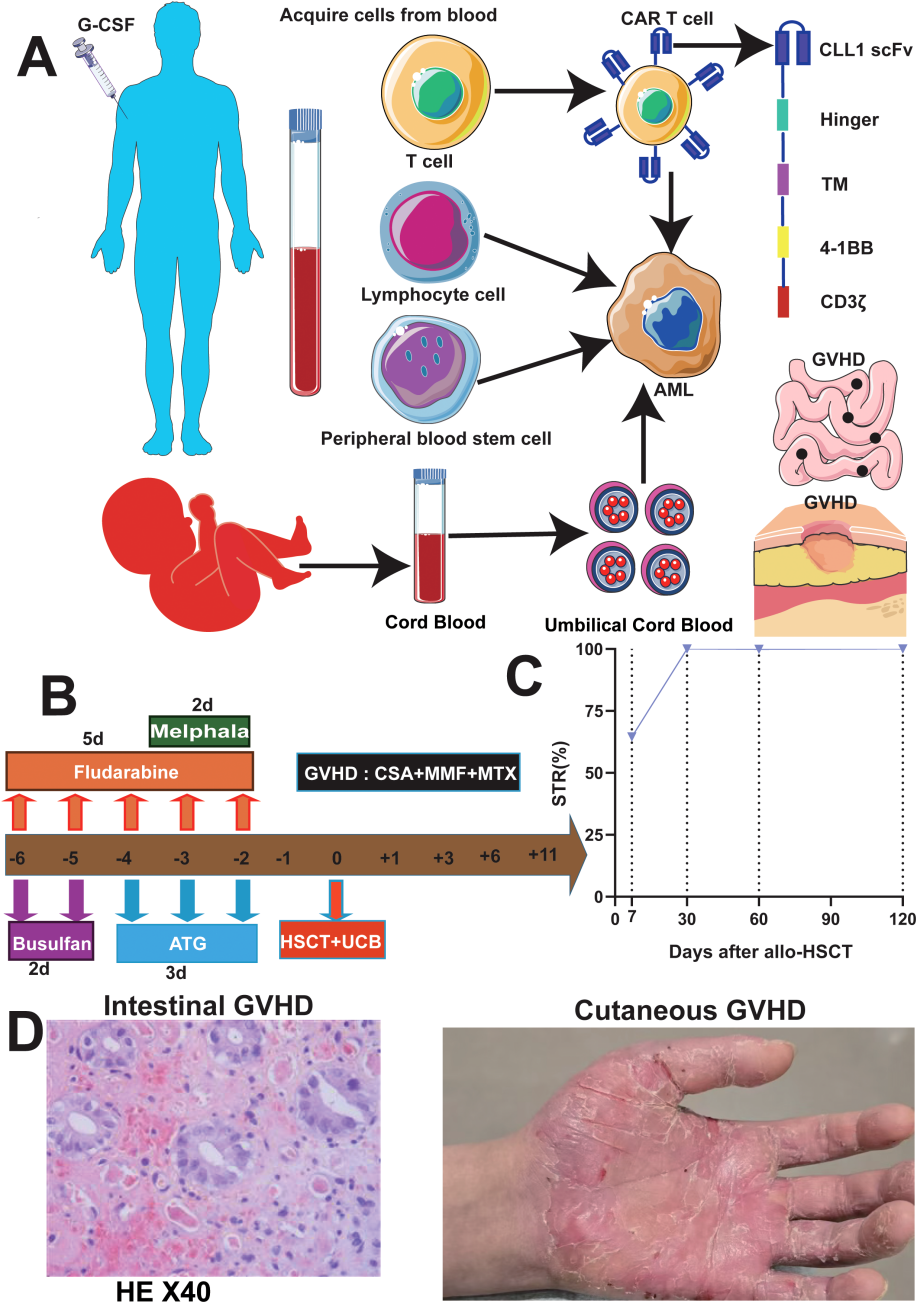
Course of treatment and development of GVHD in the patient. **(A)** patient’s course of CAR-CLL1, DLI and HSCT. **(B)** Outlines the reduced-intensity conditioning regimen for allogeneic hematopoietic stem cell transplantation, including Melphalan, Fludarabine, and Busulfan. **(C)** Demonstrates post-transplant chimerism rate variations monitored via short tandem repeat (STR) analysis. **(D)** shows that the patient developed intestinal and cutaneous GVHD, although the patient is currently in complete remission. G-CSF, granulocyte colony-stimulating factor; TM, transmembrane. GVHD, graft-versus-host disease; AML, acute myeloid leukemia; HSCT, hematopoietic stem cell transplantation; DLI, donor lymphocyte infusion, anti-thymocyte globulin; CSA, cyclosporin; MMF,Mycophenolate Mofetil; MTX, Methotrexate.

Additionally, the patient underwent repeat NGS, which revealed a new ETV6 mutation alongside the previously documented mutations (**Supplementary Table S3**). The patient experienced multiple relapses, and treatment had reached an impasse. CAR-T therapy emerged as a potential therapeutic option. Testing revealed that 97.4% of the patient’s AML cells expressed CLL1 (**Figure 3**), prompting the decision to pursue CLL1 CAR-T therapy. We generated CLL1 CAR-T cells from the peripheral blood mononuclear cells (PBMCs) of the patients. Prior to the infusion of CLL1 CAR-T cells, patients underwent a lymphodepletion regimen comprising cyclophosphamide and fludarabine. On June 28, 2024, CLL1 CAR-T cells were administered at a dose of 1×10^6^/kg. One month following CLL1 CAR-T therapy, the patient experienced disease relapse with no recovery of hematopoietic function.CAR-CLL1 therapy was attempted as a bridge to allo-HSCT but proved ineffective, with post-treatment myeloblasts at 30% and MRD 23.5% (**Supplementary Figure S1**). Moreover, 27% of CLL1+AML cells remained after CAR-CLL1 treatment (**Supplementary Figure S2**). The patient subsequently developed severe pancytopenia requiring transfusion support and later experienced septic shock (fever of 41°C, altered consciousness) due to klebsiella pneumoniae bacteremia. Following multidisciplinary consultation, the patient underwent PBSCT with UCB augmentation two months after CLL1 CAR-T treatment (**Figure 4A**). The conditioning regimen included melphalan(100mg/m^2^, -3d to -2d), fludarabine (30mg/m^2^, -6d to -2d), busulfan(0.8mg/kg, -6d to -5d), and anti-thymocyte globulin (ATG) (2.5mg/kg, -4d to -2d), with graft-versus-host disease (GVHD) prophylaxis using cyclosporine(CSA)(1.5 mg/kg, intravenous infusion every 12 hours, starting from -1d), mycophenolate mofetil(MMF)(1.0 g/d, starting from -1d), and methotrexate(MTX)(+1d 15 mg/m²,+3d,+6d,+11d 10 mg/m²) (**Figure 4B**). Two months after completing CLL1 CAR-T therapy (August 29, 2024), the patient underwent a PBSCT (brother-to-brother, male donor to male recipient, HLA-matched at 8/10, A-negative donor to A-positive recipient, CD34+ cells 4.23×10^6^/kg) combined with an UCB transplant (HLA-matched at 4/6, CD34+ cell 2.8×10^5^/kg). The patient first received the UCB infusion, followed 6 hours later by the peripheral blood stem cell infusion. Neutrophil engraftment occurred on day +14, and platelet engraftment on day +16. Bone marrow evaluation at day +30 confirmed complete remission (**Figure 2F**). By day +60, however, blasts recurred (myeloblasts 3.5%, MRD 16.92%, **Figure 2G**). Given the high risk of relapse, DLI was initiated at a dose of 1×10^6^ cells/kg per administration, delivered every 6 weeks. As of the follow-up date (April 1, 2025), a total of two infusions had been administered. The UCB played an adjunct role in this patient, with successful engraftment achieved by PBSCs. The chimerism rate was 65% at day +7 post-transplant, and subsequent peripheral blood analysis demonstrated 100% chimerism, indicating complete donor chimerism (**Figure 4C**). The patient remains in sustained remission (**Figure 2H**). The patient developed only mild GVHD post-transplant, with grade 2 skin GVHD occurring 10 days after DLI and grade 2 gastrointestinal GVHD emerging by day 14 post-DLI (**Figure 4D**). Both events were manageable. As of the follow-up date (April 1, 2025), the patient has remained in continued remission of the disease and is alive.

## Discussion

AML is a malignant hematologic tumor. For R/R AML, especially for patients who have failed CAR-T therapy, the prognosis is very poor and there is no effective treatment (5). Such patients have a huge clinical demand. At present, most CAR-T therapies for AML are in the clinical trial stage, and target selection and efficacy optimization are the core issues. At present, the main targets of CAR-T therapy for AML are CD123, CD33, CLL1 and CD7 (3, 6). CLL1 is a marker for predicting relapse in AML (7). CAR-CLL1 therapy also shows great therapeutic potential in R/R AML, and CAR-CLL1 bridging allo-HSCT is a treatment option for R/R AML (8).This case highlights a novel salvage strategy for CAR-T-refractory R/R AML using UCB, PBSCT, and DLI. Despite developing cutaneous and intestinal GVHD, the patient attained long-term leukemia-free survival, offering a potential salvage pathway for AML patients progressing after CAR-T therapy. The treatment process highlights the rescue potential of transplantation strategies for CAR-T-refractory patients while overcoming challenges including post-CAR-T immune depletion, infection crises, and donor limitations (9). The findings address a critical unmet need in hematology—salvage strategies for CAR-T-resistant AML—and resonate with global efforts to improve outcomes in R/R AML.

This represents the first reported case of PBSCT combined with UCB and DLI following CAR-CLL1 therapy failure. Although UCB transplantation and DLI are established options for R/R AML treatment, no previous cases have documented concurrent administration of these interventions. Prior case reports have described CLL1 CAR-T bridging to transplantation in R/R AML (10) and CLL1 CAR-T treatment for post-transplant AML relapse (8), both utilizing donor-derived CLL1 CAR-T cells. However, the therapeutic effect might be limited due to the impaired function of the patient’s T cells, which is also the reason for the failure of CLL1 CAR-T in this case. Notably, those patients ultimately underwent repeat transplantation when possible to maximize survival. Distinctively, our patient received autologous CLL1 CAR-T cells rather than donor-derived products. A relevant case report described an AML patient who relapsed within six months after undergoing haploidentical PBSCT combined with unrelated UCB and prophylactic DLI, with HLA loss identified as the likely mechanism of relapse (11). The unique significance of this case lies in addressing the therapeutic dilemma posed by CAR-T-induced immune collapse and severe bone marrow suppression, which typically compromise hematopoietic microenvironments and lead to frequent implantation failures or fatal infections in conventional salvage transplants (4). Following CAR-CLL1 progression, the patient successfully underwent combined UCB and PBSCT with subsequent DLI, which induced graft-versus-leukemia (GVL) effects (12). This indicates that allo-HSCT-mediated immune reconstruction may overcome resistance mechanisms associated with CAR-T therapy, potentially targeting tumor antigens not covered by CAR-T. Additionally, DLI-induced “immune reactivation” could further eliminate MRD (13). In AML patients experiencing relapse post-transplantation, DLI may salvage survival through its potent GVL effect (14). This report describes a case of MRD -positive relapse occurring 2 months post-transplantation. Following DLI treatment, the patient achieved remission again, although DLI triggered manageable GVHD. To mitigate severe GVHD risk, we maintained a low DLI dose without escalation and adhered to a 6-week infusion interval. This case underscores that DLI, driven by donor T cell-mediated GVL activity, demonstrates significant antileukemic efficacy in AML patients with post-transplant relapse (3), particularly those with early relapse or MRD positivity. Future strategies should prioritize integrating precision immune monitoring, novel combination therapies (such as DLI with hypomethylating agents or targeted drugs), and advanced cellular engineering approaches (such as gene-edited T cells) to optimize therapeutic outcomes while minimizing toxicity, ultimately enhancing long-term prognosis for AML patients (15). The potential reasons for early post-transplant relapse in this patient may include: 1. The patient underwent allo-HSCT during active AML relapse without sequential chemotherapy prior to the preconditioning regimen. 2. Insufficient GVL effect during the first month post-transplantation. 3. Prior exposure to multiple lines of therapy and multiple relapses, leading to potential resistance of AML to current treatments. 4. Clonal evolution of AML, marked by the emergence of a new ETV6 mutation and aberrant expression of CD antigens at relapse. 5. Co-occurring CEBPA and WT1 mutations, along with NRAS mutation, which may contribute to a poor prognosis and relapse propensity (16).

The GVL of allo-HSCT targets a wide range of antigens, which reduces the risk of immune escape through antigen loss or downregulation. Autologous CAR-T therapy has been engineered to target a single tumor-associated antigen, and the heterogeneity and clonal evolution of AML will increase the risk of recurrence. Allo-HSCT plays a stronger role in T cells, while autologous CAR-T may have weaker ability to kill tumors due to abnormal immune function in AML patients. The GVL effect of allo-HSCT may be longer, while the GVL effect of autologous CAR-T is shorter. The need for repeated infusions may prolong GVL. For R/R AML, both allo-HSCT and CAR-T therapy appear to be potential options. However, patients who fail both allo-HSCT and CAR-T currently lack effective treatments, and many clinical trials may exclude this population. Thus, preventing relapse in these patients remains a critical challenge and a future direction in clinical management. Based on our case report, we propose that even after allo-HSCT, high-risk AML patients with multiple relapses should receive consolidation therapies to prevent recurrence, particularly DLI (17). DLI should be initiated early—ideally starting as early as 1 month post-transplant and no later than 2 months—given our patient’s relapse at 2 months post-transplant. Fortunately, timely DLI achieved re-remission in this case. These observations highlight the importance of proactive, post-transplant immunomodulatory strategies to bridge the gap until novel therapies become available.

The potential effects of UCB transplantation in our case mainly lie in promoting the smooth implantation of PBSCT on the one hand, and on the other hand in its strong GVL effect to minimize the recurrence after allo-HSCT as much as possible. UCB was selected for its potent GVL effects and lower chronic GVHD risk, with DLI enhancing GVL-mediated deep remission (18). The dynamic balance between GVHD and GVL proved clinically consequential although cutaneous/intestinal GVHD occurred (19), its temporal correlation with MRD clearance implies that alloreactive responses might eliminate CAR-T-resistant clones. In this case report, the patient with critical disease progression had no available matched related or unrelated donors. Following the failure of CAR CLL1 therapy, we opted for salvage allo-HSCT. The patient’s cryopreserved stem cells (stored for approximately 6 months with 92% cell viability) were utilized for transplantation. To enhance engraftment potential, we incorporated UCB co-transplantation as a synergistic strategy. While conventional UCB transplantation faces challenges such as delayed engraftment and hematopoietic reconstitution, our dual approach (PBSCT + UCB) substantially mitigates these limitations. Previous studies have demonstrated superior outcomes with PBSCT plus UCB transplantation compared to bone marrow-derived stem cells combined with PBSCT transplantation. Based on this evidence, we elected to proceed with PBSCT supplemented with UCB for this patient. The combination of PBSCT with UCB, compared to marrow with PBSCT, may have contributed to improved safety and efficacy in this case (20). Temporal alignment of GVHD onset with MRD clearance suggests alloreactivity contributed to leukemia control. Peripheral blood-cord blood transplantation may offer safer engraftment than marrow-cord strategies. The intervention successfully harnessed GVL effects, highlighting the balance between toxicity and therapeutic efficacy in transplant immunology. Our study has several limitations: 1. This is a single-case report, and the findings need validation through large-scale clinical studies to further explore the generalizability of DLI efficacy in post-transplant relapse settings. 2. The follow-up duration for this patient remains relatively short, necessitating prolonged observation to assess long-term outcomes and relapse-free survival. 3. For R/R AML post-transplantation, novel and more effective therapeutic strategies (such as combination immunotherapies, targeted agents, or engineered cellular therapies) still require rigorous investigation to address unmet clinical needs.

This case challenges the conventional salvage options for CAR-T-resistant AML and proposes a feasible pathway for patients with limited donor availability (Rh-negative compatibility and cryopreserved grafts). This case demonstrates the feasibility of UCB combined with PBSCT and DLI for CAR-T-refractory R/R AML. Controlled GVHD-associated GVL effects provided therapeutic benefits even in the context of dual immune-hematopoietic failure, enabling successful engraftment and long-term survival.

## Data Availability

The original contributions presented in the study are included in the article/**Supplementary Material**. Further inquiries can be directed to the corresponding author.
