# Supplementary material for: Case Report: Combined umbilical cord blood and peripheral blood stem cell transplantation with donor lymphocyte infusion for R/R AML post CAR-CLL1 failure
Source: Front Immunol. 2025 Jun 5;16:1598754. doi: 10.3389/fimmu.2025.1598754 (PMC12176548; doi:10.3389/fimmu.2025.1598754)
Supplement: Supplementary file 1 [file Table1.docx]

**Supplementary Information**

**
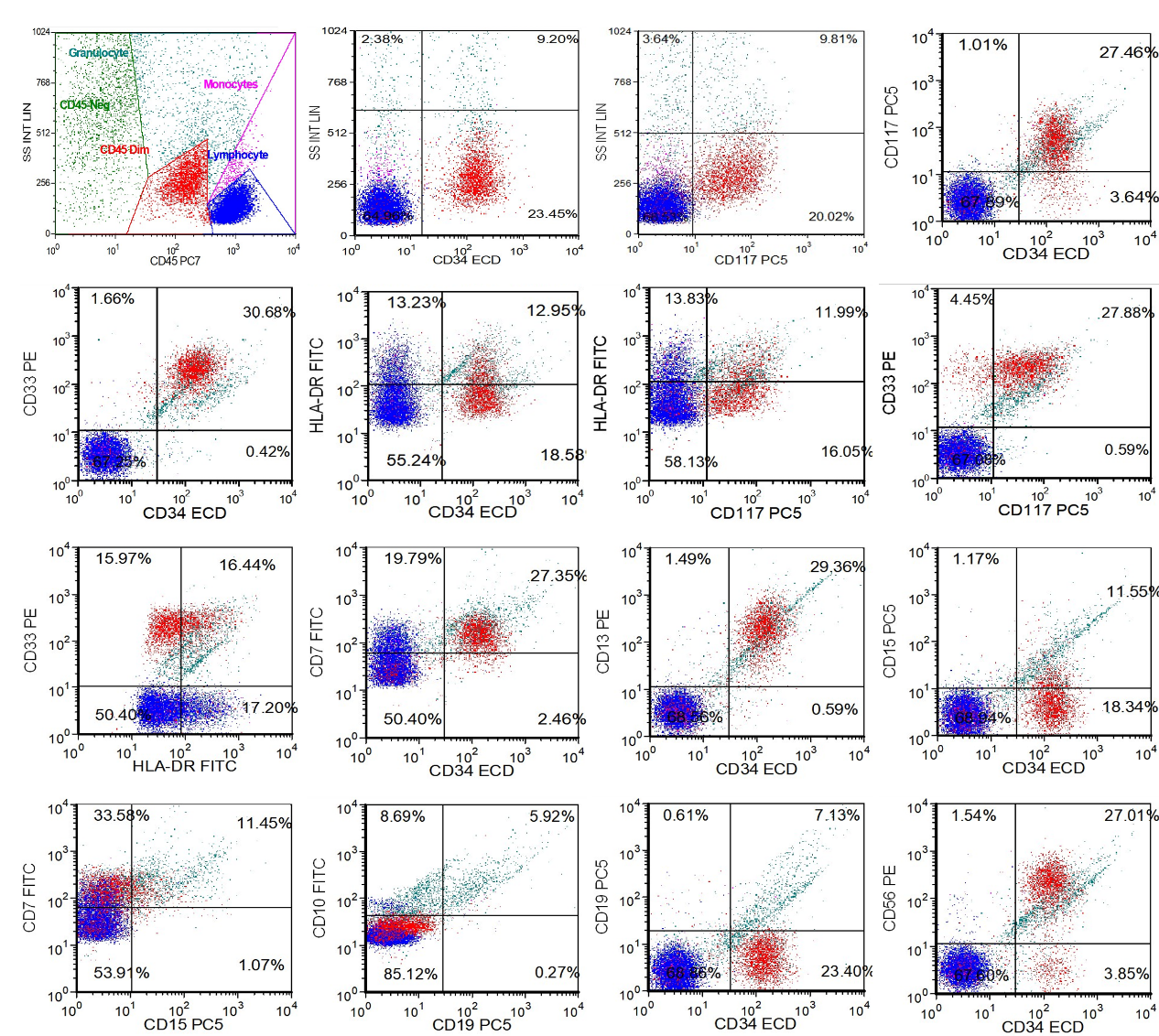
**

**Supplementary figure 1. One month after the end of CAR-CLL1 treatment, the results of flow cytometry of recurrence were reviewed.**

The results of flow cytometry showed that CD34+ cells accounted for about 23.5% of the total nuclear cells. The immunophenotype of CD34+ cells were CD34+, CD117+dim, CD33+, HLA-DR+ part, CD15+ small amount, CD13+, CD19-, CD56+ part, CD7+.About 23.5% of AML tumor cells were found in the samples


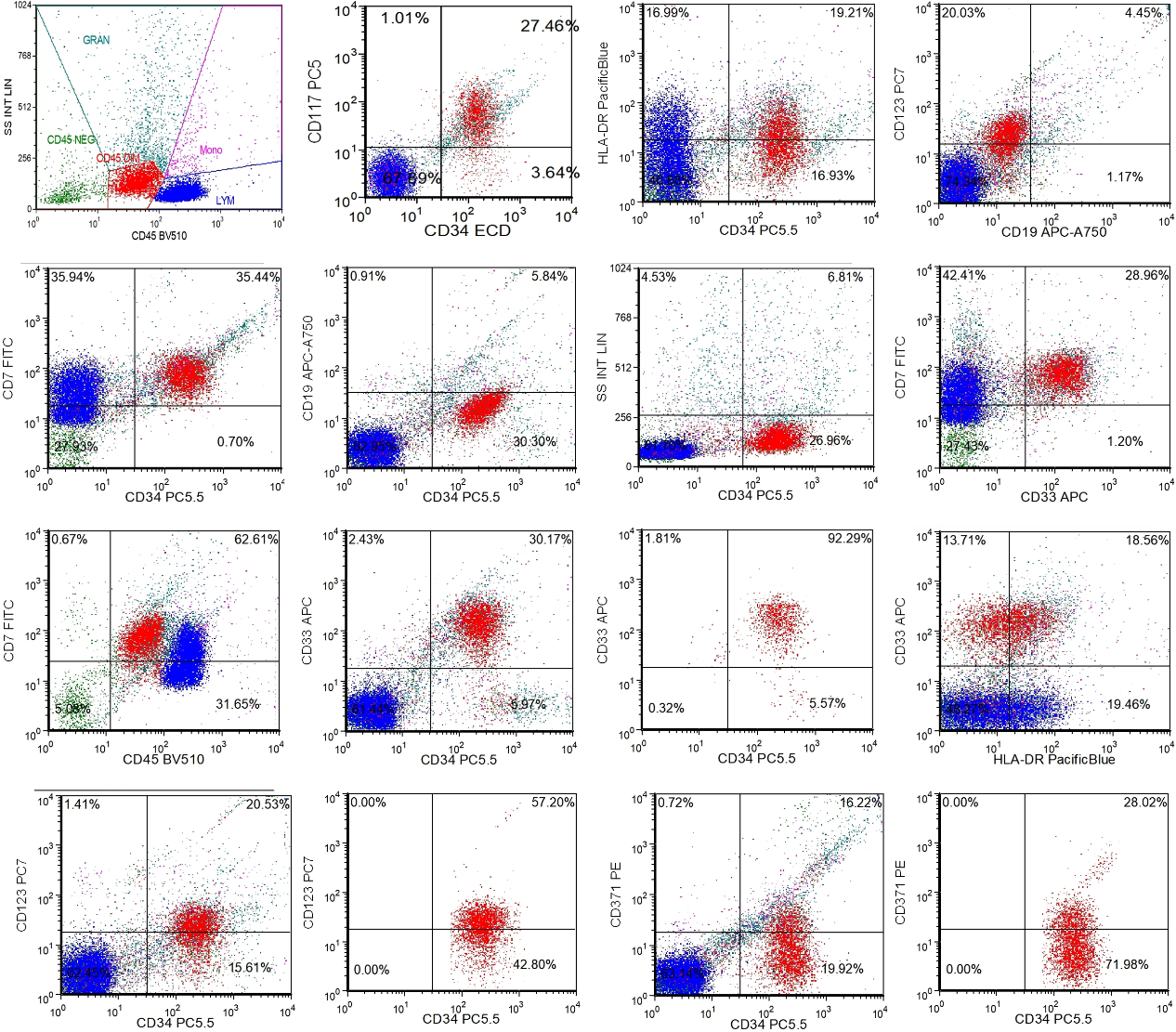


**Supplementary figure 2. CLL1+AML was detected by flow cytometry after CAR-CLL1 treatment.**

Approximately 27.0% of the myeloid blasts (tumor cells) in the submitted specimen were CD34+. The relevant CD results are as follows:

CD371+ in part, CD123+ dim, HLA-DR+ in part, CD7+, CD33+, CD19-, CD45+ dim, CD117+ dim.CD371+ cells accounted for approximately 28.0% of the total CD34+ cells.CD123+ cells accounted for approximately 57.2% of the total CD34+ cells.CD33+ cells accounted for approximately 92.3% of the total CD34+ cells.

**Supplementary Table 1. 56 genes strongly associated with myeloid tumors.**

| 56 genes strongly associated with myeloid tumors | | | | | | | |
| --- | --- | --- | --- | --- | --- | --- | --- |
| ABL1 | ARID2 | ASXL1 | ASXL2 | BCOR | BCORL1 | CALR | CBL |
| CDC25C | CEBPA | CSF3R | CSNK1A1 | DDX41 | DNMT3A | ETNK1 | ETV6 |
| EZH2 | FLT3 | GATA2 | GNAS | GNB1 | IDH1 | IDH2 | JAK2 |
| JAK3 | KDM6A | KIT | KRAS | MPL | NF1 | NPM1 | NRAS |
| PHF6 | PIGA | PPM1D | PRPF8 | PTPN11 | RUNX1 | SAMD9 | SAMD9L |
| SBDS | SETBP1 | SETD2 | SF1 | SF3B1 | SH2B3 | SRSF2 | STAG2 |
| STAT3 | TET1 | TET2 | TP53 | U2AF1 | UBA1 | WT1 | ZRSR2 |

**Supplementary Table 2. Results of NGS.**

| Mutated gene | Chromosome | Transcript ID | Mutation site | Nucleotide alteration | Mutation frequency | Sequencing depth |
| --- | --- | --- | --- | --- | --- | --- |
| CCEBPA | 19q13.11 | NM_0004364 | exon1 | c.958959ins39 | 47.70% | 1149X |
| CEBPA | 19q13.11 | NM_0004364 | exon1 | c.245dupT | 49.50% | 204X |
| NRAS | 1p13.2 | NM_002524 | exon2 | c.35G>A | 7.20% | 2615X |
| NRAS | 1p13.2 | NM_002524 | exon2 | c.34G>A | 0.90% | 2618X |
| GATA2 | 3q21.3 | NM_032638 | exon5 | c.1085G>A | 1.20% | 1578X |
| GATA2 | 3q21.3 | NM_032638 | exon5 | c.1084C>G | 0.70% | 1563X |
| WT1 | 11p13 | NM_024426 | exon7 | c.1157delC | 0.60% | 2107X |
| WT1 | 11p13 | NM_024426 | exon9 | c.1355 G>7 | 2.30% | 1972X |
| WT1 | 11p13 | NM_024426 | exon8 | c.1349 A>G | 1.90% | 1801X |

**Supplementary Table 3. Results of the second NGS.**

| Mutated gene | Chromosome | Transcript ID | Mutation site | Nucleotide alteration | Mutation frequency | Sequencing depth |
| --- | --- | --- | --- | --- | --- | --- |
| CCEBPA | 19q13.11 | NM_004364 | exon1 | c.958959ins39 | 41.26% | 3611X |
| CEBPA | 19q13.11 | NM_004364 | exon1 | c.245dupT | 47.76% | 1610X |
| NRAS | 1p13.2 | NM_002524 | exon2 | c.35G>T | 9.1% | 2373X |
| ETV6 | 12p13 | NM_001987 | exon7 | c.1199A>G | 45.91% | 2455X |
| WT1 | 11p13 | NM_024426 | exon8 | c.1349A>G | 48.50% | 1895X |
| WT1 | 11p13 | NM_024426 | exon9 | c.1355 G>T | 43.29% | 1631X |
